# The Efficacy and Safety of Ischemic Stroke Therapies: An Umbrella Review

**DOI:** 10.3389/fphar.2022.924747

**Published:** 2022-07-22

**Authors:** Yongbiao Li, Ruyi Cui, Fangcheng Fan, Yangyang Lu, Yangwen Ai, Hua Liu, Shaobao Liu, Yang Du, Zhiping Qin, Wenjing Sun, Qianqian Yu, Qingshan Liu, Yong Cheng

**Affiliations:** ^1^ Key Laboratory of Ethnomedicine of Ministry of Education, School of Pharmacy, Center on Translational Neuroscience, Minzu University of China, Beijing, China; ^2^ Institute of Chinese Materia Medica, China Academy of Chinese Medical Sciences, Beijing, China; ^3^ The People’s Hospital of Xin Tai City (Nephropathy Department), Beijing, China; ^4^ Institute of National Security, Minzu University of China, Beijing, China

**Keywords:** ischemic stroke, clinical trial, systematic review, umbrella review, neurological functional

## Abstract

**Background:** Ischemic stroke is a leading cause of morbidity and mortality in neurological diseases. Numerous studies have evaluated the efficacy and safety of ischemic stroke therapies, but clinical data were largely inconsistent. Therefore, it is necessary to summarize and analyze the published clinical research data in the field.

**Objective:** We aimed to perform an umbrella review to evaluate the efficacy and safety of ischemic stroke therapies.

**Methods:** We conducted a search for meta-analyses and systematic reviews on PubMed, the Cochrane Library, and the Web of Science to address this issue. We examined neurological function deficit and cognitive function scores, quality of life, and activities of daily living as efficacy endpoints and the incidence of adverse events as safety profiles.

**Results:** Forty-three eligible studies including 377 studies were included in the umbrella review. The results showed that thrombolytic therapy (tPA; alteplase, tenecteplase, and desmoteplase), mechanical thrombectomy (MTE), edaravone with tPA, stem cell-based therapies, stent retrievers, acupuncture with Western medicines, autologous bone marrow stromal cells, antiplatelet agents (aspirin, clopidogrel, and tirofiban), statins, and Western medicines with blood-activating and stasis-dispelling herbs (NaoShuanTong capsule, Ginkgo biloba, Tongqiao Huoxue Decoction, Xuesaitong injection) can improve the neurological deficits and activities of daily living, and the adverse effects were mild for the treatment of ischemic stroke. Moreover, ligustrazine, safflower yellow, statins, albumin, colchicine, MLC601, salvianolic acids, and DL-3-n-butylphthalide showed serious adverse events, intracranial hemorrhage, or mortality in ischemic stroke patients.

**Conclusion:** Our study demonstrated that tPA, edaravone and tPA, tPA and MTE, acupuncture and Western medicines, and blood-activating and stasis-dispelling herbs with Western medicines are the optimum neurological function and activities of daily living medication for patients with ischemic stroke.

**Systematic Review Registration**: https://inplasy.com/, identifier [INPLASY202250145].

## Introduction

Ischemic stroke is a major cause of death and disability, so prevention and effective treatment of stroke are of utmost importance in China and the West. The World Health Organization has suggested that an incidence of stroke occurs once every 5 s worldwide, approximately one-third of strokes are fatal, and another third leave survivors with permanent disability (Donkor, 2018). Moreover, surviving stroke patients impose a heavy medical burden on families and communities (Go et al., 2014). However, little is known about the efficacy and safety of treatments of ischemic stroke in the hyper-acute (0–24 h) and acute phases (1–7 days) and recovery period (>7 days) post-stroke in humans (Marzolini et al., 2019). The key challenge in the treatment of stroke is to identify the most effective way to implement the efficacious interventions currently available.

Some evidence supports national guidelines recommending the use of recombinant tissue plasminogen activator (tPA) thrombolysis for the treatment of hyperacute ischemic stroke, which can significantly improve neurological deficits (Li et al., 2017; Zhou et al., 2020). In addition, the guidelines also recommend antithrombotic (including antiplatelet and anticoagulant therapy), neuroprotection, traditional Chinese medicine, statins, and control of high-risk factors for secondary prevention of ischemic stroke (Practice, 2021). Additionally, as a bradykinin B1 and B2 receptor agonist, HUK provides functional benefits (Patel and McMullen, 2017). Furthermore, other neuroprotective drugs are supported by comprehensive clinical reports that demonstrate their efficacy and safety in improving cognitive impairment or other major domains (Practice, 2021).

Attempts to many systematic reviews and meta-analyses have been conducted to analyze the different stroke treatments. These studies, however, did not provide comprehensive appraisals of stroke therapies, and some results are still conflicting (Wu et al., 2007). A review of the latest literature, having removed repeated studies and research involving complications, followed by a meta-analysis to derive at pooled prevalence, was needed. Therefore, the present study aimed to perform an umbrella review of the systematic reviews and meta-analyses of stroke therapies through a comprehensive and updated literature search and to reach a definitive conclusion by integrating all available meta-analyses to identify which of the commercially available treatments for ischemic stroke patients are efficacious and safe.

## Materials and Methods

Our study was performed in accordance with the standard guidelines of Preferred Reporting Items for Systematic reviews and Meta-analysis (PRISMA) (Moher et al., 2009). The protocol for this review was prospectively registered at INPLASY PROTOCOL (INPLASY202250145).

### Search Strategy and Quality Assessment

A systematic search of published peer-reviewed English language literature was conducted using PubMed, Web of Science, and the Cochrane Library until March 2022. The database search terms were as follows: (Ischemic stroke) and (systematic review or meta-analysis) and clinical trial. We included meta-analyses and systematic reviews that determined the efficacy and safety of treatments in patients with stroke. Inclusion criteria were: 1) written in English; 2) published systematic review or meta-analyses; 3) including any evaluation of clinical assessment scales for stroke; 4) published in peer-reviewed journals. Studies were excluded if 1) unpublished studies; 2) no necessary sample data; 3) patients were diagnosed with other strokes; 4) the study reported insufficient details and other outcomes; and 5) the study presented the risk of bias/study limitations.

The AMSTAR2 tool was used to evaluate systematic reviews and meta-analyses (Shea et al., 2007; De Santis et al., 2021). The methodological quality of the studies was determined by the percentage of AMSTAR2 score. The percentage of AMSTAR2 score was classified into 0–33%, 34–66%, and 67%–100% indicating low quality, medium quality, and high quality, respectively.

We searched for related articles using keywords and filtering titles, and two investigators screened the literature independently. Articles were downloaded and the abstracts screened using inclusion criteria, deleting any irrelevant or repetitive articles. Thereafter, we manually searched the reference lists of the chosen studies for any other relevant studies not found in our initial search. Finally, a full-text search was performed to extract and then analyze the data from articles.

### Data Extraction

According to the following criteria, three investigators (Yongbiao Li, Ruyi Cui, and Fangcheng Fan.) independently selected those trials that met the inclusion criteria. The main characteristics of the selected study were extracted in a table including the year of publication, study design, number of studies, and regimens for the treatment. We included results evaluating the efficacy of drugs in patients with at least one of the clinical assessment scales: 1) the incidence of intracranial hemorrhage (sICH); 2) the primary outcomes included: global neurological deficit scores such as the National Institutes of Health Stroke Scale (NIHSS) score ≤1 and the Neurological Function Deficit Scores (NFDS); 3) all-cause mortality; 4) dependence assessed by Barthel Index (BI) scores ≥95; 5) modified Rankin Scale score of 0–1 or return to baseline (mRS); 6) clinical effect, defined according to the nationally approved criteria, is divided into essentially recovered, significant improvement, improvement, no change, deterioration, and death (the first three categories are judged to be effective); 7) the secondary outcomes included the following: cognitive function scoring; related hemorheology and lipid metabolism outcomes; quality of life; and 8) incidence of adverse events (AE). The selection of assessments was extracted on study size, sample size, mean difference (Fixed, 95% CI) or odds ratio (Fixed, 95% CI), and heterogeneity (I^2^). A percentage of 0–25% was classified as mild, 26–50%, as moderate, and 51–75%, as significant between-study heterogeneity. If I^2^ > 50%, a random-effects model was used for the analysis, or the data were analyzed on the fixed-effects model (Wang et al., 2016).

### Statistical Analysis

The sample size and mean difference were used to calculate the four clinical assessment scales. NIHSS/mRS/BI scores were used to evaluate neurological status, and behavioral symptoms in patients were calculated by NFDS. We focused on the clinical effect is divided into essentially recovered, significant improvement, no change, deterioration; cognitive function scoring; quality of life as activities of daily living. All data analyses were performed by GraphPad Prism 5.0 software. The results were expressed as OR ± SD (standard deviation). The adverse events have assessed the incidence of adverse events, and the OR was calculated. Therefore, mean difference or odds ratio with 95% CI and *p* values were used to assess the efficacy and safety of the study medications.

## Results

**FIGURE 1:**
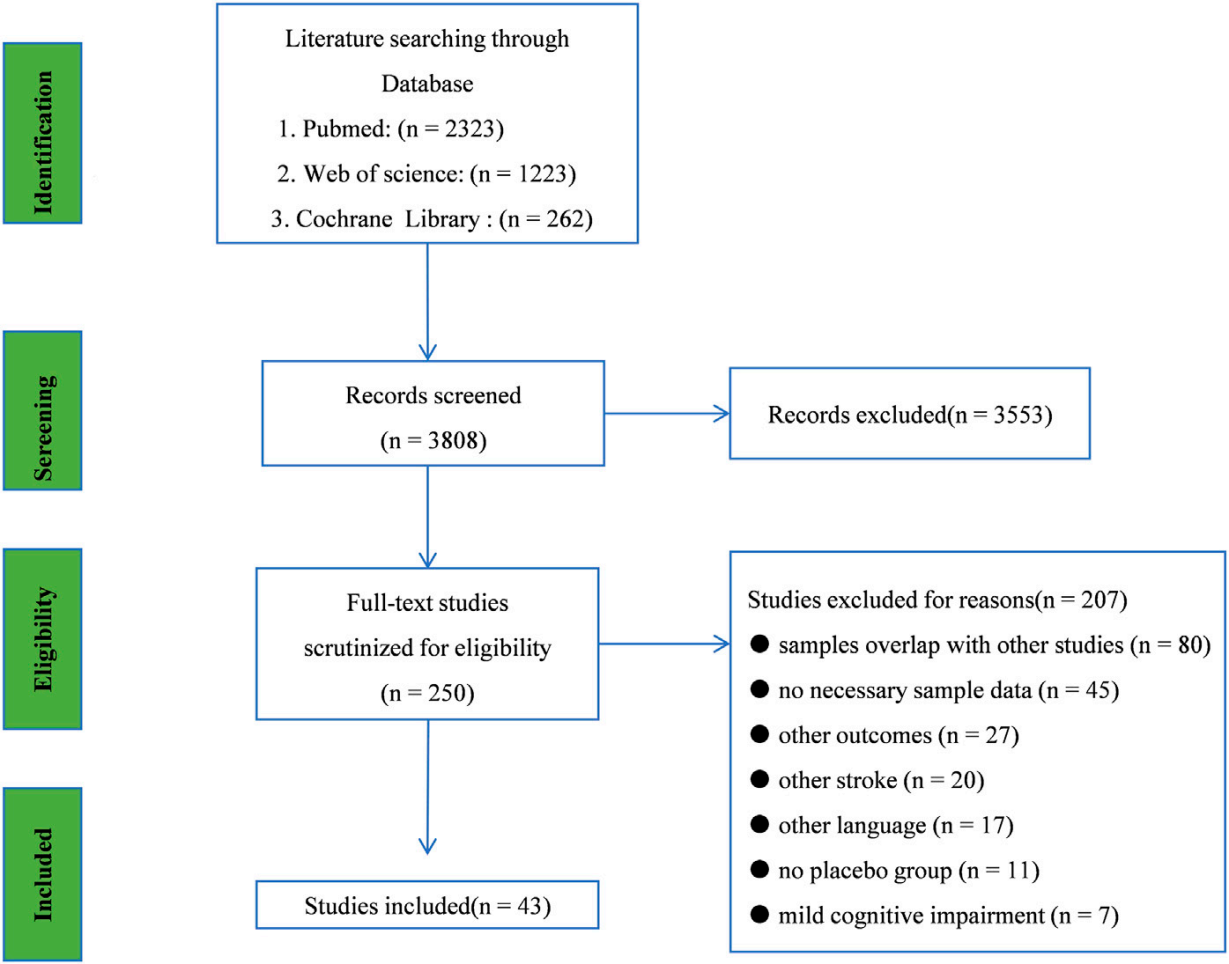
Searching and screening process: literature search and study selection Through the initial search, we retrieved a total of 3,808 records from PubMed, Web of Science, and Cochrane Library. After examining the titles and abstracts, 250 studies were selected for further full-text scrutiny. In all, 207 studies were excluded due to the following reasons: sample overlap with other studies (*n* = 80), no necessary sample data (*n* = 45), other outcomes (*n* = 27), other stroke (*n* = 20), other language (*n* = 17), no placebo group (*n* = 11), and mild cognitive impairment (*n* = 7).

Literature search and study selection through the initial search, we retrieved a total of 3,808 records from PubMed, Web of Science, and Cochrane Library. After examining the titles and abstracts, 250 studies were selected for further full-text scrutiny. In all, 207 studies were excluded due to the following reasons: samples overlap with other studies (*n* = 80), no necessary sample data (*n* = 45), other outcomes (*n* = 27), other stroke (*n* = 20), other language (*n* = 17), no placebo group (*n* = 11), mild cognitive impairment (*n* = 7), (Figure 1). Thus, 43 studies were included in the umbrella review: Pan et al., 2020; Pan et al., 2020); Liu et al., 2021); (Liu et al., 2011); Blann et al., 2015); (Blann et al., 2015); Emberson et al., 2014) (Emberson et al., 2014); Peng et al., 2014); (Peng et al., 2014); Zhang et al., 2019); Shang et al., 2019); Puñal-Riobóo et al., 2015; Yuan et al., 2008; (Fu et al., 2013), Fan et al., 2014), Lin et al., 2014); Xu et al., 2015); Cao and Li, (2015); Marmagkiolis et al., 2015); Zheng et al., 2017), Li et al., 2017), Zhang et al., 2017), Chong et al., 2020); (Zhao et al., 2021), Li et al. (2020); (Li et al., 2020), Gao et al., 2021); Huang et al., 2020); (Huang and Xiao, 2021), Liu et al., 2022), Feng et al., 2021), Lee et al. (2010); Zhou et al., 2022); Hu et al., 2021); Hong and Lee, 2015, Liu et al., 2021); (Liu et al., 2021), Xin et al., 2020); Katsanos et al., 2020), Wang et al., 2021), Liu et al., 2019; (Liu et al., 2019), Xu et al., 2019); (Zhang et al., 2019), (Huang et al., 2020), Yang et al., 2015), Yang et al., 2015), (Ni et al., 2020); (Thelengana et al., 2019), Shi et al., 2014) and (Siddiqui et al., 2013), (Liu et al., 2016), Kaesmacher et al., 2019); (Kaesmacher et al., 2019), Li et al., 2014). The main characteristics, bias analysis, and the quality scores of the included studies are shown in Table 1 and Supplementary material.

**TABLE 1:** Description and AMSTAR2 scores of included studies.

Study	Condition	Studies included	Study duration (median, range)	Daily dose (median, range)	Outcome	AMSTAR2 score	Study quality
Ni et al. (2013)	Ligustrazine versus placebo	3	14w (2w–48w)	240 mg/day	1. Effect and 2. sICH	5/11	low
Xin et al. (2020)	Heparin versus Placebo	9	12w	<40 mg/day	1. mRS, 2. NIHSS, 3. sICH, 4. DOS, and 5. AE	7/11	middle
Shang et al. (2019)	MTE versus placebo	7	12w	NA	1. mRS and 2. sICH	8/11	high
Kaesmacher et al. (2019)	tPA plus MTE versus placebo	12	12w	NA	1. mRS and 2. sICH	9/11	high
Li et al. (2014)	Acupuncture plus XM versus placebo	17	12W	NA	1. Effect	8/11	high
Liu et al. (2021)	Nimodipine versus placebo	8	18w (12w–24w)	NA	1. Effect, and 2. NFDs	10/11	high
Blann et al. (2015)	Aspirin plus clopidogrel versus placebo	24	12w	60 mg/day	1. Effect and2. sICH	9/11	high
Emberson et al. (2014)	tPA versus placebo	12	3 h (0–6 h)	<0.85 mg/kg/day	1. Effect, 2. sICH, and 3. NIHSS	10/11	high
Peng et al. (2014)	XNJ versus placebo	13	4w	45 ml (30–60 ml/day)	1. Effect, 2. NFDs, and 3. AE	6/11	middle
Zhang et al. (2019)	NST versus placebo	13	12w	50 mg/day	1. Effect, 2. NFDs, 3 .BI, and 4. mRS	10/11	high
Yuan et al. (2008)	Chuanxiong versus Placebo	3	24w (1w–48w)	120 mg (80–160 mg/day)	1. NFDs and 2. AE	10/11	high
Fu et al. (2013)	XXMT versus placebo	8	12w (4w–24w)	NA	1. NIHSS, 2. mRS, and 3. Effect	5/11	low
Fan et al. (2014)	Safflower yellow versus placebo	7	2w	50 mg/day	1. Effect, 2. NFDs, and 3. AE	5/11	low
Lu et al. (2014)	Rhubarb versus placebo	12	2w (1w–4w)	NA	1. Effect, 2. NFDs, 3. BI, 4. NIHSS, and 5. AE	6/11	middle
Xu et al. (2015)	WD versus placebo	13	2w (2w–4w)	NA	1. Effect, 2. sICH, and 3. NFDS	4/11	low
Cao and Li. (2015)	MSCs versus placebo	5	3w (1w–6w)	5 × 10^7^-2.6 × 10^8^ cell	1. NIHSS, 2. mRS, 3. BI, and 4. AE	6/11	middle
Marmagkiolis et al. (2015)	stent retrievers versus placebo	5	12w	NA	1. mRS, 2. sICH, and 3. AE	8/11	high
Zheng et al. (2017)	Puerarin versus placebo	16	1w (1w–2w)	300 mg (100–500 mg/day)	1. Effect and 2. NFDs	6/11	middle
Li et al. (2017)	Alpha1 versus placebo	6	6 h (3–9 h)	90 mg/kg/day	1. Effect, 2. sICH, and 3. AE	8/11	high
Zhang et al. (2017)	Cerebrolysin versus placebo	7	12w (1w–12w)	50 ml/day	1. mRS, 2. BI, and 3. AE	9/11	high
Chong et al. (2020)	Ginkgo biloba versus placebo	12	12w (1w–12w)	100 mg (40–160 mg)/day	1. NIHSS, 2. NFDs, 3. sICH, and 4. AE	9/11	high
Li et al. (2020)	Stem cell-based versus placebo	9	12w (1w–12w)	5 × 10^6^-2.97 × 10^9^ cell	1. NIHSS, 2. mRS, 3. BI, and 4. AE	9/11	high
Zhou et al. (2020)	tirofiban versus placebo	6	18w (12w–24w)	(0.1-0.4 ug/kg/day)	1. Effect, 2. sICH, and 3. AE	5/11	low
Gao et al. (2021)	BHD versus placebo	11	16w (8w–24w)	NA	1. Effect, 2. NIHSS, and 3. AE	9/11	high
Huang and Xiao. (2021)	Albumin versus placebo	4	15w (2w–48w)	1.3 mg (0.6–2 mg/kg/day)	1. Effect	9/11	high
Liu et al. (2022)	DZSM versus placebo	28	7w (1w–13w)	NA	1. mRS, 2. NFDs, 3. BI, and 4. NIHSS	10/11	high
Feng et al. (2021)	XST plus XM versus placebo	12	2w (2w–4w)	NA	1.Effect and 2. NIHSS	5/11	middle
Lee et al. (2010)	Intra-A versus placebo	5	12w	NA	1. mRS, 2. BI, and 3. NIHSS	5/11	middle
Zhou et al. (2022)	TQHX plus XM versus placebo	12	4w	NA	1. Effect and 2. NFDs	9/11	high
Hu et al. (2021)	Edaravone plus rt-PA versus placebo	17	2w (1w–4w)	60 mg/day	1. sICH and 2. NIHSS	5/11	middle
Hong and Lee. (2015)	Statins versus placebo	18	6w (1w–12w)	8 mg/kg/day	1. Effect and 2. NFDs	9/11	high
Liu et al. (2021)	ZL versus placebo	7	2w	1.4 mg (1.2–1.6 g/day)	1. mRS, 2. BI, and 3. NIHSS	7/11	middle
Xin et al. (2020)	salvianolic acids versus placebo	12	2w (1w–4w)	200 mg (100–300 mg/day)	1. Effect, 2. NIHSS, 3. mRS, and 4. BI	4/11	low
Katsanos et al. (2020)	Colchicine versus placebo	4	74w (4w–144w)	0.5 mg/day	1. AE	3/11	low
Liu et al. (2019)	ANP versus placebo	18	2w	3 g/day	1. Effect, 2. NIHSS, and 3. NFDs	9/11	high
Xu et al. (2015)	NBP versus placebo	12	6w (1w–12w)	100 mg/day	1. BI, 2. NIHSS, and 3. AE	9/11	high
Wang et al. (2021)	Pntsp versus placebo	20	6w (2w–10w)	470 mg (140–800 mg/day)	1. NIHSS, 2. mRS, 3. BI, and 4. AE	10/11	high
Huang et al. (2020)	HUK versus placebo	16	3 h (0–6 h)	0.15 PNA	1. NIHSS, 2. NFDs, and 3. AE	7/11	middle
Yang et al. (2015)	Mailuoning versus Placebo	21	12w	204 mg (8–400 mg/day)	1. Effect, 2. NFDs, 3. BI, 4. NIHSS, and 5. AE	9/11	high
Ni et al. (2020)	Cinepazide maleate versus placebo	4	7w (2w-12w)	320 mg/day	1. mRS, 2. BI, and 3. AE	7/11	middle
Thelengana et al. (2019)	TNK versus placebo	4	3 h (0–6 h)	0.15 mg (0.1–0.2 mg/kg/day)	1. Effect, 2. NFDs, 3. BI, 4. NIHSS, and 5. AE	9/12	high
Shi et al. (2014)	Cilostazol versus placebo	6	30w (1w-60w)	690 mg (80–1300 mg/day)	1. sICH and 2. AE	10/11	high
Siddiqui et al. (2013)	MLC601 versus placebo	2	13w (2w-24w)	405 mg (10–800 mg/day)	1. NFDs and 2. BI	5/11	low

**FIGURE 2:**
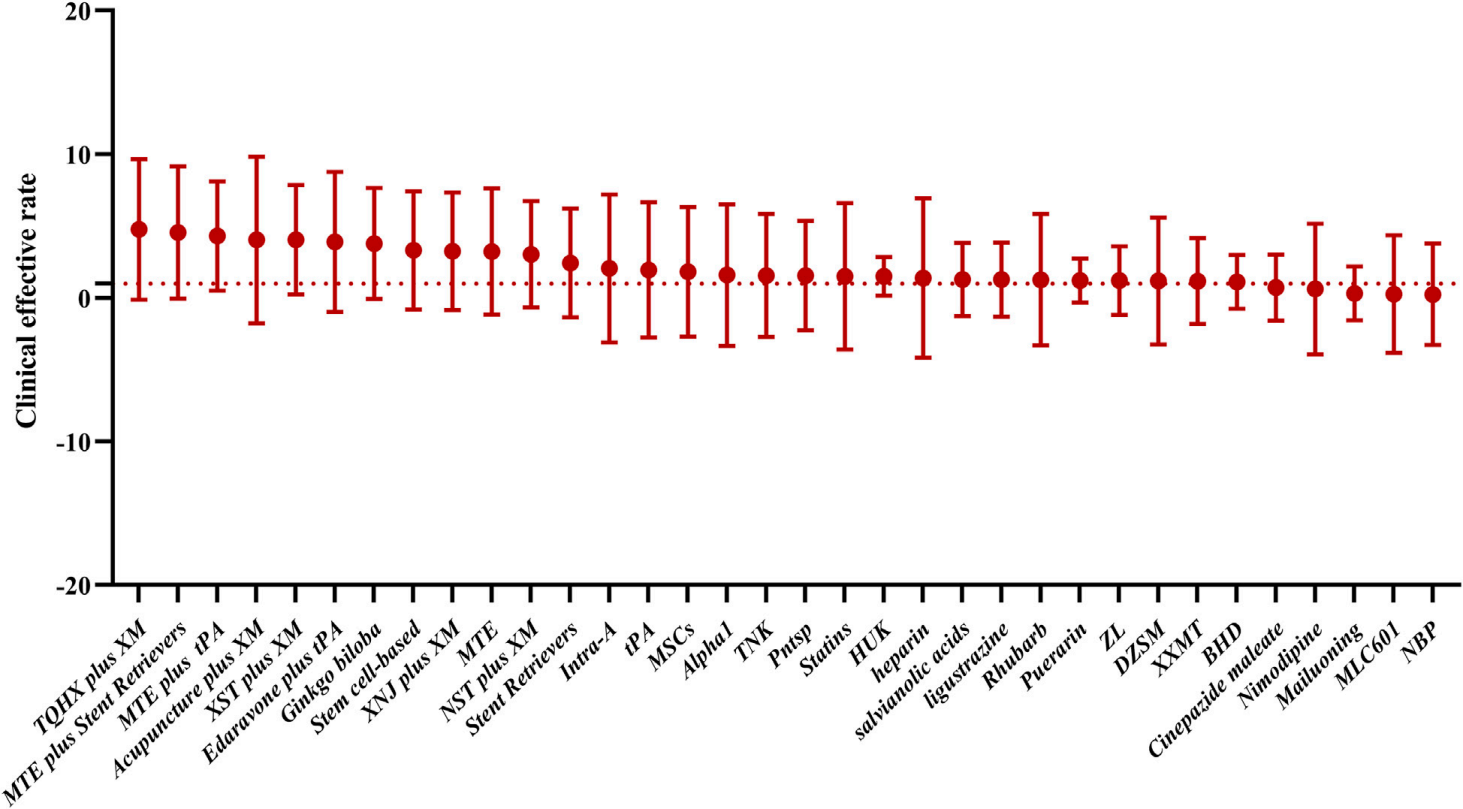
Total clinical efficacy was used to evaluate the effect of drug therapy on ischemic stroke. In this study, the possible order of efficacy of the drugs was TQHX plus XM, MTE plus stent retrievers, MTE plus tPA, acupuncture plus XM, XST plus XM, edaravone plus tPA, Ginkgo biloba, stem cell-based therapy, XNJ plus XM, MTE, NST plus XM, stent retrievers, intra-A, tPA, MSCs, Alpha1, TNK, Pntsp, statins, HUK, heparin, salvianolic acids, ligustrazine, rhubarb, puerarin, ZL, DZSM, XXMT, BHD, cinepazide maleate, nimodipine, Mailuoning, MLC601, and NBP.

As shown in Table 1, a total of 377 clinical trials were included, with 43 drug therapies in the treatment groups. All studies were randomized controlled clinical trials, and the treatment duration ranged from 1 to 72 weeks. In total, 24 meta-analyses included were of high quality according to AMSTAR2 score, 12 meta-analyses included were of middle quality according to AMSTAR2 score, and seven meta-analyses included were of low quality according to AMSTAR2 score. The total clinical efficacy was used to evaluate the effect of drug therapy on ischemic stroke (Figure 2).

### Clinical Effect

Clinical effective rate was observed in 18 studies. Detailed characteristics of included studies are listed in Table 2. The clinical effect of ligustrazine (OR: 1.28, 95% CI: 1.10–1.50), nimodipine (OR: 0.62, 95% CI: 0.50–0.78), aspirin plus clopidogrel (OR: 1.82, 95% CI: 1.08–2.57), tissue plasminogen (tPA) (RR: 1.95, 95% CI: 1.10–2.56), Wen Dan Decoction (WD) (OR: 1.60, 95% CI: 1.43–1.79), Xingnaojing capsule and Western medicines (XNJ) (OR: 3.25, 95% CI: 2.30–4.59), NaoShuanTong capsule plus Western medicines (NST plus XM) (OR: 3.04, 95% CI: 1.76–5.26), Xiaoxuming decoction (XXMT) (OR: 1.17, 95% CI: 1.09–1.26), Rhubarb (OR: 1.27, 95% CI: 1.18–1.37), stem cell-based (OR: 3.31, 95% CI: 2.54–4.31), puerarin (RR: 1.22, 95% CI: 1.17–1.28), Buyang Huanwu decoction (BHD) (OR: 1.12, 95% CI: 0.99–1.27), statins (OR: 1.5, 95% CI: 1.29–1.75), salvianolic acids (OR: 1.29, 95% CI: 1.25–1.33), *Panax notoginseng* saponin (Pntsp) (RR: 1.55, 95% CI: 1.37–2.55), Xuesaitong injection plus western medicines (XST plus XM) (OR: 4.04, 95% CI: 2.86–5.73), Tongqiao Huoxue Decoction plus Western medicines (TQHX plus XM) (OR: 5.43, 95% CI: 3.77–7.82), Ginkgo biloba (RR: 3.79, 95% CI: 2.49–5.78), edaravone plus rt-PA (OR: 3.90, 95% CI: 3.02–5.02) Zhilong Huoxue Tongyu capsule (ZL) (RR: 1.2, 95% CI: 1.12–2.29), desmoteplase (alpha1) (OR: 1.59, 95% CI: 1.08–2.35), acupuncture plus XM (OR: 4.04, 95% CI: 2.93–5.57), and DZSM (Dengzhan Shengmai capsule) (OR: 1.18, 95% CI: 1.12 to 1.24) was significantly better compared with placebo. Moreover, ANP, ZL, and edaravone combined with western medicines significantly improve the total clinical effective rate compared to placebo.

**TABLE 2:** Results of pairwise meta-analyses for the clinical effect.

Comparative medication	Reference medication	Number of studies	Pairwise meta-analyses					
			Number of control	Number of patients	MD/OR/RR	95% CI	I^2^	*P*
Ligustrazine	Placebo	3	321	322	1.28	[1.10, 1.50]	NA	0.05
Acupuncture	Placebo	14	643	536	4.04	[2.93, 5.57]	0	0.00001
tPA	Placebo	4	814	804	1.95	[1.10, 2.56]	NA	0.002
Nimodipine	Placebo	8	677	806	0.62	[0.50, 0.78]	NA	0.0001
Aspirin plus clopidogrel	Placebo	12	100	100	1.82	[1.08, 2.57]	NA	0.001
XNJ	Placebo	13	431	408	3.25	[2.30, 4.59]	0	0.00001
NST	Placebo	13	246	243	3.04	[1.76, 5.26]	0	0.00001
Stem cell-based therapy	Placebo	20	950	844	3.31	[2.54, 4.31]	0	0.0001
Edaravone plus rt-PA	Placebo	15	591	591	3.90	[3.02, 5.02]	0	0.0001
XXMT	Placebo	8	242	289	1.17	[1.09, 1.26]	0	0.0001
Rhubarb	Placebo	12	350	438	1.27	[1.18, 1.37]	18	0.00001
WD	Placebo	13	3,773	3,341	1.60	[1.43, 1.79]	46	0.0001
Puerarin	Placebo	16	1,427	1,540	1.22	[1.17, 1.28]	47	0.00001
Alpha1	Placebo	6	217	222	1.59	[1.08, 2.35]	0	0.019
BHD	Placebo	11	350	334	1.12	[0.99, 1.27]	69	0.002
XST plus XM	Placebo	12	879	890	4.04	[2.86, 5.73]	NA	0.001
Ginkgo biloba	Placebo	9	417	416	3.79	[2.49, 5.78]	NA	0.0001
TQHX plus XM	Placebo	12	733	755	5.43	[3.77, 7.82]	NA	0.0001
ZL	Placebo	7	293	278	1.2	[1.12, 2.29]	0	0.0001
HUK	Placebo	9	338	338	1.30	[1.21, 1.41]	0	0.00001
Statins	Placebo	18	3,013	2,988	1.5	[1.29, 1.75]	0	0.01
Salvianolic acids	Placebo	12	1884	1893	1.29	[1.25, 1.33]	14	0.00001
Pntsp	Placebo	20	48	48	1.55	[1.37, 2.55]	0	0.0001
DZSM	Placebo	5	341	340	1.18	[1.12, 1.24]	85.7	0.0001

CI, confidence interval; MD, mean difference; OR, risk ratio; I^2^, heterogeneity, NST, NaoShuanTong capsule; XNJ, Xingnaojing capsule; XXMT, Xiaoxuming decoction; Pntsp, *Panax notoginseng* Saponin; XST, plus XM: Xuesaitong injection plus Western medicines; TQHX, Tongqiao Huoxue decoction; ZL, Zhilong Huoxue Tongyu capsule; BHD, Buyang Huanwu decoction; Alpaga1: Desmoteplase; WD, Wen Dan Decoction. Western medicines (XM) (tPA, antiplatelet agents, statins, and edaravone).

### NIHSS Score

The effects of the medications on clinical change were assessed by National Institutes of Health Stroke Scale (Table 3). Eight studies (20.0%) showed that XXMT (MD: −1.86, 95% CI: −3.25–−0.48), safflower yellow (MD: −3.42, 95% CI: −5.38–−2.98), MSCs (MD: −1.85, 95% CI: −2.77–−0.93), ZL (MD: −2.6, 95% CI: −3.41–−1.79), salvianolic acids (MD: −1.44, 95% CI: −1.97–−0.91), heparin (OR: 1.95, 95% CI: 0.74–5.11), XST (MD: −3.17, 95% CI: −4.14 to −2.20), intra-arterial fibrinolysis (Intra-A) (OR: 2.24, 95% CI: 1.27–3.95), edaravone plus rt-PA (MD: 3.95, 95% CI: 2.92–4.99), and human urinary kallidinogenase (HUK) (MD: −1.65, 95% CI, −2.12−−1.71) were significantly different compared with placebo. In contrast, DL-3-n-butylphthalide (NBP) (OR: 0.73, 95% CI: −0.14 to 1.59, *p* = 0.1), BHD (MD: 1.66, 95% CI: −1.08 to 4.40, *p* = 0.1), and DZSM (MD: 0.57, 95% CI: 0.44.0.73, *p* = 0.11) showed no change or a deterioration.

**TABLE 3:** Results of pairwise meta-analyses for the NIHSS score.

Comparative medication	Reference medication	Number of studies	Pairwise meta-analyses					
			Number of control	Number of patients	MD/OR/RR	95% CI	I^2^	*P*
Heparin	Placebo	9	260	317	1.95	[0.74, 5.11]	80	0.03
XXMT	Placebo	8	91	95	−1.86	[−3.25, −0.48]	10	0.008
Safflower yellow	Placebo	7	368	394	−3.42	[−5.38, −2.98]	82	0.004
MSCs	Placebo	5	52	57	−1.85	[−2.77, −0.93]	24	0.0001
BHD	Placebo	11	96	96	1.66	[−1.08, 4.40]	64	0.1
XST	Placebo	12	879	890	−3.17	[−4.14, −2.20]	NA	0.001
Intra-A	Placebo	5	130	204	2.24	[1.27, 3.95]	0	0.005
Edaravone plus rt-PA	Placebo	17	860	859	3.95	[2.92, 4.99]	92	0.0001
ZL	Placebo	7	115	330	−2.6	[−3.41, −1.79]	50	0.0001
Salvianolic acids	Placebo	12	435	462	−1.44	[−1.97, −0.91]	57	0.001
NBP	Placebo	12	108	108	0.73	[−0.14, 1.59]	89	0.1
HUK	Placebo	16	667	659	–1.65	[−2.12, −1.71]	84	0.00001
DZSM	Placebo	5	341	340	0.57	[0.44, 0.73]	44.2	0.11

CI, confidence interval; MD, mean difference; OR, risk ratio; I^2^, heterogeneity; rt-PA, alteplase; MSCs, autologous bone marrow stromal cells; XXMT, Xiaoxuming decoction; XST, Xuesaitong injection; NBP, DL-3-n-butylphthalide; BHD, Buyang Huanwu decoction; Intra-A, intra-arterial Fibrinolysis; HUK, human urinary kallidinogenase.

### Rankin Scale (mRS) Score

From our search, the effects of the medications on clinical change were assessed by Rankin Score (mRS) (Table 4). In total, 18 studies (42.5%) including tPA (OR: 1.31, 95% CI: 1.07–3.59), tPA plus mechanical thrombectomy (MTE) (OR: 4.32, 95% CI: 2.16–7.46), MTE (OR: 3.23, 95% CI: 1.75–7.33), stent retrievers (OR: 2.43, 95% CI: 1.91–3.09), cerebrolysin (RR: −049, 95% CI: −1.21 to 0.24), ZL (MD: −0.57, 95% CI: −0.84 to −0.30), salvianolic acids (MD: −0.88, 95% CI: −1.11–−0.64), heparin (OR: 1.38, 95% CI: 0.61–3.56) and Rhubarb (OR: 3.11, 95% CI: 2.06–4.68), Intra-A (RR: 2.05, 95% CI: 1.33–3.14), DZSM (MD: −0.75, 95% CI: −1.02–−0.48), and cinepazide maleate (MD: 0.607, 95% CI: 0.46–0.801) showed better outcomes for mRS score than placebo. The other treatments “Safflower yellow (MD: −4.18, 95% CI: −5.38–−2.98, *p* = 0.1) and MSCs (RR: 1.81, 95% CI: 0.37–8.95, *p* = 0.47)” indicated no significant difference in effectiveness as compared to placebo.

**TABLE 4:** Results of pairwise meta-analyses for the mRS score.

Comparative medication	Reference medication	Number of studies	Pairwise meta-analyses					
			Number of control	Number of patients	MD/OR/RR	95% CI	I^2^	*P*
Heparin	Placebo	12	2,145	550	1.38	[0.61, 3.56]	83	0.01
Safflower yellow	Placebo	13	368	394	−4.18	[−5.38,−2.98]	52	0.1
Rhubarb	Placebo	13	350	438	3.11	[2.06, 4.68]	18	< 0.05
MSCs	Placebo	7	86	86	1.81	[0.37, 8.95]	57	0.47
tPA	Placebo	4	814	804	1.31	[1.07, 3.59]	NA	0.01
MTE	Placebo	5	414	404	3.23	[1.75, 7.33]	NA	0.008
MTE plus stent retrievers	Placebo	5	142	143	4.56	[2.63, 7.9]	0	0.0001
tPA plus MTE	Placebo	17	2639	2640	4.32	[2.16, 7.46]	51	0.01
Stent retrievers	Placebo	5	653	634	2.43	[1.91, 3.09]	0	0.00001
Cerebrolysin	Placebo	5	971	808	−0.49	[−1.21, 0.24]	73.6	0.052
Intra-A	Placebo	12	171	224	2.05	[1.33, 3.14]	0	0.001
ZL	Placebo	9	45	60	−0.57	[−0.84, −0.30]	37	0.0001
Salvianolic acids	Placebo	7	210	242	−0.88	[−1.11, −0.64]	0	0.001
DZSM	Placebo	28	341	340	−0.75	[−1.02, −0.48]	85.9	0.0001
Cinepazide maleate	Placebo	4	236	234	0.607	[0.46, 0.801]	NA	0.0004

CI, confidence interval; MD, mean difference; OR, risk ratio; I^2^, heterogeneity; MSCs, autologous bone marrow stromal cells; NST, NaoShuanTong capsule; tPA: tissue plasminogen XNJ, Xingnaojing capsule; MTE: mechanical thrombectomy, ZL, Zhilong Huoxue Tongyu capsule; Intra-A, intra-arterial fibrinolysis.

### Barthel Index Score

The effects of the medications on clinical change were assessed by Barthel Index (BI) Score (Table 5). Ten studies (25%) showed that autologous bone marrow stromal cells (MSCs) (MD: 2.50, 95% CI: −4.69–9.68), TQHX plus XM (MD: 2.45, 95% CI: 1.16–3.73), ZL (MD: 9.75, 95% CI: 7.15–12.36), NST (MD: 8.15, 95% CI: 3.79–12.52), Intra-A (MD: 1.6, 95% CI: 1.01–2.51), DZSM (MD: 8.97, 95% CI: 5.88,12.05) and cinepazide maleate (MD: 0.719, 95% CI: 0.542, 0.956), and MLC601 (MD: 2.35, 95% CI: 1.31, 4.23) were significantly different compared with placebo. In contrast, NBP (MD: 1.65, 95% CI: 1.25–2.04), *p* = 0.08) showed no difference compared to placebo.

**TABLE 5:** Results of pairwise meta-analyses for the BI score.

Comparative medication	Reference medication	Number of studies	Pairwise meta-analyses					
			Number of control	Number of patients	MD/OR/RR	95% CI	I^2^	*P*
NST	Placebo	13	304	289	8.15	[3.79, 12.52]	75	0.0005
MSCs	Placebo	5	88	88	2.50	[−4.69,9.68]	74	< 0.05
Intra-A	Placebo	5	139	204	1.6	[1.01, 2.51]	0	0.04
TQHX plus XM	Placebo	12	225	226	2.45	[1.16, 3.73]	89	0.0001
ZL	Placebo	7	115	130	9.75	[7.15, 12.36]	0	0.001
NBP	Placebo	12	165	160	1.65	[1.25, 2.04]	67	0.08
DZSM	Placebo	5	341	340	8.97	[5.88, 12.05]	85.9	0.0001
Cinepazide maleate	Placebo	4	236	236	0.719	[0.542, 0.956]	0	0.012
MLC601	Placebo	2	237	436	2.35	[1.31, 4.23]	0	0.004

CI, confidence interval; MD, mean difference; OR, risk ratio; I^2^, heterogeneity; MSCs, autologous bone marrow stromal cells; NST, NaoShuanTong capsule; TQHX, Tongqiao Huoxue Decoction; ZL, Zhilong Huoxue Tongyu capsule; NBP, DL-3-n-butylphthalide; BHD, Buyang Huanwu decoction; Intra-A, intra-arterial fibrinolysis.

### Neurological Function Deficit Score

Table 6 presents the results of the comparisons of behavioral symptoms; a total of seven studies were assessed by NFD scores. Patients treated with XNJ (MD: −3.78, 95% CI: −4.75 to −2.81), NST (MD: 8.15, 95% CI: 10.11–49.10), Chuanxiong (MD: −3.11, 95% CI: −5.22–−1.00), Safflower yellow (MD: 3.11, 95% CI: 2.06–4.68), Rhubarb (MD: −3.36, 95% CI: −6.10–−0.62), Puerarin (MD: −3.69, 95% CI: −4.67–−2.71), Pntsp (MD: −3.36, 95% CI: −4.20–−2.53), HUK (MD, 1.30, 95% CI, 1.21 to 1.41), and Mailuoning (OR: 0.31, 95% CI: 0.23–0.42) showed better behavioral symptoms than those administered (*p* < 0.05). Moreover, Ginkgo biloba use was also associated with an improvement in activities of daily living and functional outcomes (MD: 9.52; 4.66 to 14.33, *p* < 0.001). Subgroup analysis suggests that the impact was larger when using an injectable formulation of Ginkgo biloba compared to the oral formulation. The other treatments indicated no significant difference in effectiveness as compared to placebo (*p* > 0.05) (Albumin (MD: 1.04, 95% CI: 0.85–1.27). TNK (MD: 1.56, 95% CI: 1.0–2.43), DZSM (MD: −2.81, 95% CI: 4.17–−1.44), and MLC601 (MD: 0.27, 95% CI: −0.02–0.55).

**TABLE 6:** Results of pairwise meta-analyses for NFDs.

Comparative medication	Reference medication	Number of studies	Pairwise meta-analyses					
			Number of control	Number of patients	MD/OR/RR	95% CI	I^2^	*P*
XNJ	Placebo	13	356	347	−3.78	[−4.75, −2.81]	54	0.00001
NST	Placebo	13	100	100	8.15	[10.11, 49.10]	95	0.0005
Chuanxiong	Placebo	3	80	81	−3.11	[−5.22, −1.00]	0	0.0039
Safflower yellow	Placebo	7	368	394	3.11	[2.06, 4.68]	0	0.00001
Rhubarb	Placebo	12	210	210	−3.36	[−6.10, −0.62]	89	0.00001
Puerarin	Placebo	16	659	699	−3.69	[−4.67, −2.71]	70	0.00001
Albumin	Placebo	4	804	807	1.04	[0.85, 1.27]	0	0.65
Salvianolic acids	Placebo	12	235	235	−8.65	[−11.10, −6.20]	31	0.001
Pntsp	Placebo	20	1464	1435	−3.36	[−4.20, −2.53]	74	0.0001
Nimodipine	Placebo	8	677	806	0.54	[0.50, 0.78]	NA	0.0001
HUK	Placebo	9	338	338	1.30	[1.21, 1.41]	0	0.00001
DZSM	Placebo	5	341	340	−2.81	[−4.17, −1.44]	85.9	0.1
Mailuoning	Placebo	15	736	755	0.31	[0.23, 0.42]	0	0.001
TNK	Placebo	4	656	671	1.56	[1.0, 2.43]	0	0.05
MLC601	Placebo	2	275	520	0.27	[−0.02, 0.55]	66	0.06

CI, confidence interval; MD, mean difference; OR, risk ratio; I^2^, heterogeneity, NST, NaoShuanTong capsule; XNJ, Xingnaojing capsule; Pntsp, *Panax notoginseng* Saponin; TQHX, Tongqiao Huoxue decoction; TNK, tenecteplase.

### Extracranial Hemorrhage (sICH)

The sICH events resulting from administration of other treatments were mild, and Safflower yellow (*p* = 0.93), stent retrievers (OR: 1.08, 95% CI: 0.64–2.30), Alpha1 (OR: 1.25, 95% CI: 0.97–1.62), Ginkgo biloba (OR: 0.82, 95% CI: 0.43–1.57), tirofiban (OR: 1.14, 95% CI: 0.72–1.82), heparin (OR: 0.71, 95% CI: 0.25–2.05), edaravone plus rt-PA (OR: 0.44, 95% CI: 0.29–0.66), MTE plus stent retrievers (OR: 0.59, 95% CI: 0.35–0.97), MTE (OR: 3.05, 95% CI: 0.44–21.23), MTE plus tPA (OR: 0.93, 95% CI: 0.72–1.19), TNK (OR: 1.07, 95% CI: 0.6–1.93), and cilostazol (OR: 0.29, 95% CI: 0.15–0.56) had no significant difference on sICH events between these groups and placebo groups (Table 7).

**TABLE 7:** Results of pairwise meta-analyses for extracranial hemorrhage.

Comparative medication	Reference medication	Number of studies	Pairwise meta-analyses					
			Number of control	Number of patients	MD/OR/RR	95% CI	I^2^	*P*
Heparin	Placebo	9	288	330	0.71	[0.25, 2.05]	32	0.22
Safflower yellow	Placebo	7	368	394	NA	NA	0	0.93
Stent retrievers	Placebo	5	652	634	1.08	[0.64, 2.30]	0	0.63
Ginkgo biloba	Placebo	12	266	281	0.82	[0.43, 1.57]	0	0.443
Tirofiban	Placebo	6	216	213	1.14	[0.72, 1.82]	0	0.57
Edaravone plus rt-PA	Placebo	8	221	221	0.44	[0.29, 0.66]	0	0.93
Alpha 1	Placebo	6	467	595	1.25	[0.97, 1.62]	9	0.09
TNK	Placebo	4	658	676	1.07	[0.6, 1.93]	0	0.81
MTE plus stent retrievers	Placebo	5	146	144	0.59	[0.35,0.97]	0	0.83
MTE	Placebo	5	141	140	3.05	[0.44, 21.23]	0	0.25
tPA plus MTE	Placebo	7	2639	2640	0.93	[0.72, 1.19]	29	0.13
Cilostazol	Placebo	6	1728	1731	0.29	[0.15, 0.56]	0	0.77

CI, confidence interval; MD, mean difference; OR, risk ratio; I^2^, heterogeneity, TNK, tenecteplase.

### Mortality

Fifteen studies reported all-cause mortality at the end of follow-up. Ligustrazine (OR: 1.67, 95% CI: 1.02–2.67), statins (OR: 0.85, 95% CI: 0.77–0.93) were significant different compared with placebo. In contrast, stent retrievers (OR: 0.81, 95% CI: 0.58–1.12), cerebrolysin (OR: 0.82, 95% CI: 0.55–1.22), Ginkgo biloba (OR: 1.21, 95% CI: 0.29–5.09), stem cell-based (MD: 0.6, 95% CI: 0.35–1.03), tirofiban (OR: 0.53, 95% CI: 0.13–2.07), albumin (OR: 1.1, 95% CI: 0.9–1.34), Alpha1 (OR: 1.05, 95% CI: 0.7–1.59), heparin (OR: 0.9, 95% CI: 0.74–1.09), Intra-A (OR: 0.83, 95% CI: 0.48–1.39), edaravone plus rt-PA (MD: 0.43, 95% CI: 0.13–1.42), tPA (OR: 1.04, 95% CI: 0.75–1.43), DZSM (MD: 0.54, 95% CI: 0.31–0.95), TNK (MD: 1.03, 95% CI: 0.69–1.52), and cilostazol (MD: 0.80, 95% CI: 0.42 to 1.53, *p* = 0.52) had no significant differences of mortality events between these groups and placebo groups (*p* > 0.05) (Table 8).

**TABLE 8:** Results of pairwise meta-analyses for mortality.

Comparative medication	Reference medication	Number of studies	Pairwise meta-analyses					
			Number of control	Number of patients	MD/OR/RR	95% CI	I^2^	*P*
Ligustrazine	Placebo	3	321	322	1.67	[1.02, 2.67]	95	< 0.05
Heparin	Placebo	9	2703	1145	0.9	[0.74, 1.09]	1	0.42
tPA	Placebo	4	814	804	1.04	[0.75, 1.43]	NA	0.83
Stent retrievers	Placebo	5	653	634	0.81	[0.58, 1.12]	29	0.19
Alpha 1	Placebo	6	467	595	1.05	[0.7, 1.59]	0	0.8
Cerebrolysin	Placebo	7	971	808	0.82	[0.55, 1.22]	0	0.81
Ginkgo biloba	Placebo	12	213	228	1.21	[0.29, 5.09]	43	1.8
Stem cell-based therapy	Placebo	9	218	217	0.6	[0.35, 1.03]	4	0.4
Tirofiban	Placebo	6	218	223	0.53	[0.13, 2.07]	63	0.1
Albumin	Placebo	4	1928	1938	1.1	[0.9, 1.34]	0	0.51
Intra-A	Placebo	5	171	224	0.83	[0.48, 1.39]	0	0.46
Edaravone plus rt-PA	Placebo	4	474	472	0.43	[0.13, 1.42]	0	0.87
Statins	Placebo	18	3034	3021	0.85	[0.77, 0.93]	0	0.003
DZSM	Placebo	5	341	340	0.54	[0.31, 0.95]	85.9	0.23
TNK	Placebo	4	658	676	1.03	[0.69, 1.52]	0	0.9
Cilostazol	Placebo	6	1728	1731	0.80	[0.42, 1.53]	0	0.52

CI, confidence interval; MD, mean difference; OR, risk ratio; I^2^, heterogeneity; Intra-A, intra-arterial fibrinolysis; rt-PA, alteplase; TNK, tenecteplase.

### Adverse Events

Adverse events of the meta-analysis of participants with at least one adverse event indicated a beneficial effect in favor of placebo treatment compared with salvianolic acids (OR: 1.45, 95% CI: 1.11–1.91, *p* = 0.007), Pntsp (*RR*: 0.62, 95% CI: 0.39–0.97, *p* = 0.04), colchicine (OR: 0.31, 95% CI: 0.13–0.71, *p* = 0.006), and NBP (RR: 3.55, 95% CI, 1.19 –10.56; *p* < 0:05). The adverse events resulting from administration of other treatments were mild, and Chuanxiong (OR: 1.02, 95% CI: 0.35–2.96), MSCs (RR: 0.43, 95% CI: 0.18–1.05), Cerebrolysin (OR: 1.18, 95% CI: 0.86–1.64), Ginkgo biloba (OR: 1.48, 95% CI: 0.51–2.71), Stem cell-based (MD: 2.59, 95% CI: 0.11–5.93), TQHX (OR: 1.78, 95% CI: 0.51–6.2), HUK (RR: 0.01, 95% CI: 0.02–0.04), Mailuoning (OR: 1.39, 95% CI: 0.28–6.76), and cinepazide maleate had no significant differences in adverse events between these groups and placebo groups (*p* > 0.05) (Table 9). Among all of the trials, in the HUK groups, six cases of hypotension, four cases of fever, two cases of flushing, two cases of vomiting, one case of headache, one case of arrhythmia, and one case of pruritus were reported. In addition, no deaths and four serious adverse events were reported in the MLC601 group.

**TABLE 9:** Results of pairwise meta-analyses for AE.

Comparative medication	Reference medication	Number of studies	Pairwise meta-analyses					
			Number of control	Number of patients	MD/OR/RR	95% CI	I^2^	*P*
Chuanxiong	Placebo	3	50	49	1.02	[0.35, 2.96]	NA	0.09
MSCs	Placebo	5	64	44	0.43	[0.18, 1.05]	0	0.06
Cerebrolysin	Placebo	7	971	808	1.18	[0.86, 1.64]	23	0.27
Ginkgo biloba	Placebo	12	388	406	1.48	[0.51, 2.71]	54	0.07
Stem cell-based therapy	Placebo	9	136	139	2.59	[0.11, 5.93]	0	0.87
TQHX plus XM	Placebo	12	180	180	1.78	[0.51, 6.2]	0	0.36
Salvianolic acids	Placebo	12	1496	1498	1.45	[1.11, 1.91]	0	0.007
Colchicine	Placebo	4	2764	2788	0.31	[0.13, 0.71]	0	0.006
NBP	Placebo	4	108	108	3.55	[1.19, 10.56]	0	< 0.05
Pntsp	Placebo	20	361	354	0.62	[0.39, 0.97]	0	0.04
HUK	Placebo	9	387	387	0.01	[0.02, 0.04]	0	0.50
Mailuoning	Placebo	2	64	65	1.39	[0.28, 6.76]	0	0.57
cinepazide maleate	Placebo	NA	648	643	NA	NA	NA	0.82

CI, confidence interval; MD, mean difference; OR, risk ratio; I^2^, heterogeneity; MSCs, Pntsp, *Panax notoginseng* Saponin; autologous bone marrow stromal cells; TQHX, Tongqiao Huoxue Decoction.

## Discussion

Our umbrella review was conducted on the data derived from treatments for ischemic stroke patients, which was used to appraise the relative effectiveness and safety of therapies. We attempted to summarize data from published systematic reviews and meta-analyses to find if there are significant beneficial treatments for ischemic stroke patients. Our study showed that thrombolytic therapy (rt-PA, TNK, and alpha1), MTE, stem cell-based therapies, stent retrievers, acupuncture plus XM, MSCs, antiplatelet agents (aspirin, clopidogrel, and tirofiban), statins, and blood-activating and stasis-dispelling herbs can improve the neurological deficits and activities of daily living in patients with ischemic stroke. MTE plus Stent Retrievers or tPA, TQHX plus XM, XST plus XM, and NST plus XM show better clinical efficacy and safety. Ligustrazine, safflower yellow, statins, Pntsp, albumin, HUK, colchicine, MLC601, salvianolic acids, and NBP have no important impact on neurological deficits or activities of daily living. In addition, tPA, MTE, stem cell-based therapies, Stent Retrievers, Acupuncture, NST, Ginkgo biloba, TQHX, XST, and XNJ show no serious adverse events in ischemic stroke patients. Our results need to be interpreted with caution to determine the optimal treatment strategy for ischemic stroke patients.

The effects of tPA may be considerable for ischemic stroke which is incurable with current treatment paradigms, and other medications that may slow down the progression of ischemic stroke patients are worth exploring. Previous studies have showed that tPA or MTE has beneficial effects on hyperacute period ischemic stroke (Thelengana et al., 2019) (Liu et al., 2016), while one study demonstrated that tPA plus MTE performed best (Kaesmacher et al., 2019). Our results indicated that all tPA, MTE, MTE plus tPA, MTE plus Stent Retrievers, TQHX plus XM, XST plus XM, and NST plus XM were more effective for neurological function or activities of daily living compared with placebo. Researches have demonstrated that there was a higher effect of Stent Retrievers and MTE observed for acute ischemic stroke than that observed for the mild ischemic stroke patients (Punal-Rioboo et al., 2015). Similar to these studies, Stent Retrievers and MTE treatment showed statistically significant improvement in clinical effect compared to placebo in our study. Research studies have demonstrated that Human serum albumin has shown remarkable efficacy in rodent models of ischemic stroke (Huang and Xiao, 2021). Unfortunately, our study has demonstrated that showing no statistically significant difference between the albumin and control groups (*p* > 0.05). Considering pulmonary edema and other complications are more likely to occur in such patients after albumin infusion, the administration of albumin therapy for acute ischemic stroke should be carried out with utmost caution.

The behavioral symptoms of patients with ischemic stroke are often evaluated by NFDS/NIHSS/BI/mRS, which assesses the severity and frequency of neuropsychiatric symptoms. As a result, previous meta-analyses have reported that the efficacy of blood-activating and stasis-dispelling herbs may be related to the severity of ischemic stroke. In addition, tPA, MTE plus tPA, MTE plus Stent Retrievers, blood-activating and stasis-dispelling herbs plus XM was reported as only a modest but significant effect found on behavior in ischemic stroke patients (Peng et al., 2014; Punal-Rioboo et al., 2015; Kaesmacher et al., 2019; Shang et al., 2019; Zhang et al., 2019). In our study, Alpha1 was more effective for neurological improvement rate compared with placebo. Unfortunately, the lack of placebo controls in NFDS/NIHSS/BI/mRS score studies may limit their validity. Interestingly, MSCs are not significant in mRS score but significant in NIHSS/BI score. Moreover, nimodipine can significantly improve clinical outcomes compared with placebo, although it does not significantly reduce the incidence rate of recurrent hemorrhage and adverse reactions. In addition, tPA and MTE affected mRS scores and was recommended by the FDA. We considered treatment with ligustrazine, Safflower yellow albumin, MLC601, ANP, rhubarb, and NBP to not affect neurological deficits and activities of daily living because of the lack of statistical significance of results. Patients with ischemic stroke deteriorate progressively with varying degrees of severity of disease, which may affect the results obtained from pooling data. Moreover, measurement time after dosing can affect NFDS/NIHSS/BI/mRS scoring results and cause them to be biased.

Previous meta-analyses have demonstrated that patients treated with intra-arterial fibrinolysis provided a modest and better improvement in clinical effect change (Roaldsen et al., 2022). In addition, drug combination shows a statistically significant advantage compared to placebo the short-term and long-term analysis. Although the effect of single blood-activating and stasis-dispelling herbs (TQHX, NST, XST, etc.) use is not ideal (Erratum, 2017), they show a modest and better effect in combination with XM (Wu et al., 2007). Furthermore, ischemic stroke agents are likely to have an important effect on increasing neurological function or activities of daily living in mild to moderate ischemic stroke patients. In this study, the quality evaluated by AMSTAR2 scores of systematic reviews of ligustrazine, safflower yellow, cerebrolysin, BHD, salvianolic acids, and ZL was low, and these may not have an important impact on neurological function or activities of daily living. First, ischemic stroke is a sudden disease, our review mainly selected clinical studies to demonstrate short-term efficacy on neurological function. Although long-term clinical trials are ethically questionable, those that are high-quality are essential to uncover comparative differences between treatments of ischemic stroke. Second, we believe that further analyses are needed to clarify the factors associated with the increased placebo effect over time in global clinical trials. In the treatment of ischemic stroke, the safety of the treatments is critical since they should be taken on a long-term basis. The number of participants with at least one serious adverse event such as nausea, diarrhea, cardiovascular, gastrointestinal, and other disorders was extracted. Previous meta-analyses have demonstrated that acute and convalescent stroke patients treated with antiplatelet agents showed a modest improvement, although there is a risk of intracranial hemorrhage (Zhou et al., 2020). In this review, edoxaban was likely to provide more protection from stroke and sICH than placebo, aspirin alone, or aspirin plus clopidogrel in both clinical trials and unselected community populations. Moreover, statins were found to be effective for primary and secondary prevention of ischemic stroke in the study through the aggressive reduction of cholesterol. Some studies have found that using statins before an ischemic stroke can increase collateral circulation and improve prognosis. Despite an increased risk of bleeding conversion, thrombolytic use of statins resulted in overall improvement. Recent studies have also found statins to be associated with atrial fibrillation. In addition, the promotion of collateral circulation by neuroprotective drugs may be related to the induction of NO synthesis and angiogenesis in vascular endothelium (Hu et al., 2021). In addition, the incidence of withdrawals due to adverse events tended to be higher in the salvianolic acids albumin, MLC601, and NBP treatment than in placebo groups. Moreover, our study summarized that MTE, stem cell-based therapies, stent retrievers, acupuncture plus XM, NST, Ginkgo biloba, TQHX, XST, and XNJ show no serious adverse events in ischemic stroke patients.

In recent years, stem cell-based therapies (MSCs, stem cell-based) as a treatment to investigate ischemic stroke patients has been a potential therapy (Cao and Li, 2015; Li et al., 2020). A previous study has shown that Intra-A results in a better beneficial effect for cognition and activities of daily living (Lee et al., 2010). Similar to these studies, stem cell-based therapies may show effectiveness for neurological deficits and activities of daily living in this study. However, clinical trials of stem cell-based therapies for ischemic stroke are still in the early stage. Many factors such as cell types, cell numbers, delivery routes, time windows, and medical and rehabilitation therapies affect the efficacy of stem cells. Well-designed RCTs are necessary to explore the benefit of stem cell-based therapies as treatment in patients with ischemic stroke, and further research effects should be carefully explored.

In general, the treatment for patients with ischemic stroke is aimed at promoting independence, clear embolism, maintaining function, and treating symptoms. Previous meta-analyses and reviews have focused on the possible effectiveness and safety of stem cell-based therapies, stent retrievers, acupuncture, MSCs, antiplatelet agents, statins, and blood-activating and stasis-dispelling herbs (Li et al., 2014; Cao and Li, 2015; Punal-Rioboo et al., 2015; Shang et al., 2019; Zhang et al., 2019; Li et al., 2020; Zhao et al., 2021; Zhou et al., 2022), even though patients experience modest efficacy and many adverse events with the treatment. As a result, we need to identify an efficacious and safe treatment paradigm for ischemic stroke patients. Studies have shown that MTE plus tPA, MTE plus Stent Retrievers, TQHX plus XM, XST plus XM, NST plus XM, and acupuncture plus XM improved neurological deficits and activities of daily living, and the adverse effects were mild for the treatment of ischemic stroke (Li et al., 2014; Kaesmacher et al., 2019; Shang et al., 2019; Li et al., 2020; Shen et al., 2020). However, a larger sample size and long-term follow-up studies are needed to find the reliability of this medication. Due to tPA, MTE, tPA plus edaravone, blood-activating, and stasis-dispelling herbs plus XM efficacy in improving neurological deficits and activities of daily living, we believe that tPA or tPA plus other drugs can be employed as first-line treatment.

### Limitations

The limitations to this study should be acknowledged. First, direct comparative evidence of treatments for ischemic stroke patients in our included studies was limited. Second, other factors may have led to the umbrella review inconsistencies, such as the duration and quality of studies. Furthermore, a considerable number of studies could not be included as they did not have the abovementioned data.

## Conclusion

In conclusion, our study suggested that tPA, tPA plus MTE, acupuncture plus XM, tPA plus edaravone, and blood-activating and stasis-dispelling herbs plus XM are the optimum cognitive and activities of daily living medication for patients with ischemic stroke. In the future, the combination of well-tolerated agents and other significant beneficial treatments should be used for patients with ischemic stroke, which will contribute to the successful construction of a similar study.

## Data Availability

The original contributions presented in the study are included in the article/Supplementary Material; further inquiries can be directed to the corresponding author.
