# Supplementary material for: The Efficacy and Safety of Ischemic Stroke Therapies: An Umbrella Review
Source: Front Pharmacol. 2022 Jul 22;13:924747. doi: 10.3389/fphar.2022.924747 (PMC9355553; doi:10.3389/fphar.2022.924747)

First of all, in terms of the funnel plot, the effect values of various mental health problems are concentrated above the graph and evenly distributed on both sides of the total effect, which preliminarily shows that the problem of publication bias is not obvious. Second, regarding the results of Egger's linear regression: the regression intercept for medical treatment of ischemic stroke was −0.31, 95% CI [−1.89, 1.26], P = 0.09. The results indicated that there was no significant publication bias in this study. In addition, since there are many articles on the treatment of ischemic stroke with a single drug, we selected articles from high-cited and high-level journals.(Fig1)

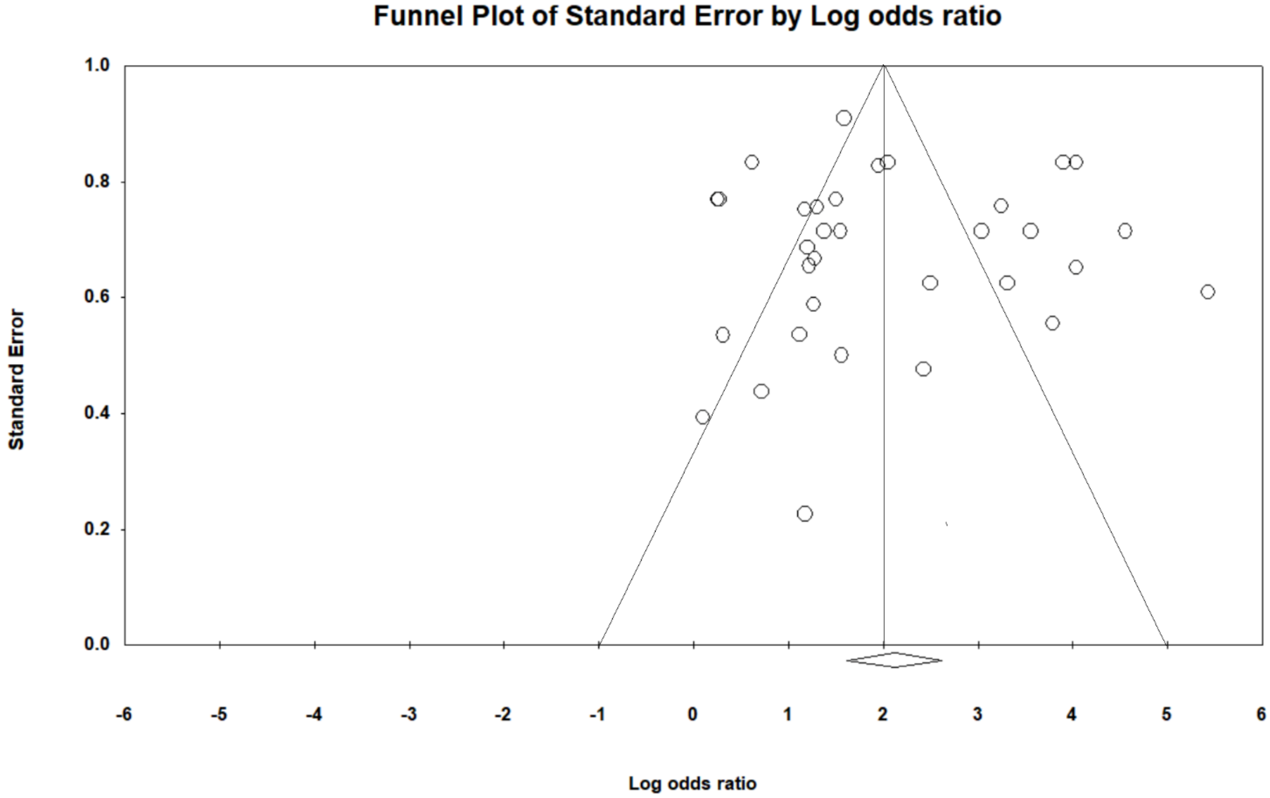

Supplement: Supplementary file 1 [file Datasheet1.PDF]
